# On Kiestein

**Published:** 1851-11

**Authors:** 


*On Kiestein.* By Dr. Veit.—In consequence of the discrepancy of opinion which prevails among observers as to to the value to be attached to the appearance of the urine termed Kiestein, as diagnostic of pregnancy, Dr. Veit has, during a year and a half, been conducting a series of experiments at the Halle Lying-in-Institution. He has examined for this purpose the urine of 10 men, of 4 non-pregnant females, and of 48 women in various stages of pregnancy. He comes to the same conclusion as Höfle (*Chemie und Mikroskop am Krankenbette*) and, recently, Lehmann, vix., that the so-called pellicle of Kiestien is no peculiar matter at all, and is not of the slightest value as a sign of pregnancy. In urine of both non-pregnant and pregnant women, pellicles are formed, containing vibrones, and frequently the triple phosphate; the chief difference between the respective urines being, that in that of pregnant women, alkaline, and in that of non-pregnant women, acid, reaction more frequently manifests itself. This may in some measure depend upon the greater concentration of the urine in pregnancy, and the larger proportion of mucus mixed with it, as a consequence of the changes induced in the condition of the mucous membrane of the bladder by the passive hyperæmia of that organ during pregnancy. Persons partaking of a more nitrogenous diet than did the poor pregnant women whose urine was examined, might furnish different results in this respect.—*Brit. and For. Review, Oct.* 1851, *from Zeitsch. für Geburt.*

